# Boosting the Biocontrol Efficacy of *Bacillus amyloliquefaciens* DSBA-11 through Physical and Chemical Mutagens to Control Bacterial Wilt Disease of Tomato Caused by *Ralstonia solanacearum*

**DOI:** 10.3390/microorganisms11071790

**Published:** 2023-07-12

**Authors:** Dhananjay Kumar Yadav, Venkatappa Devappa, Abhijeet Shankar Kashyap, Narendra Kumar, V. S. Rana, Kumari Sunita, Dinesh Singh

**Affiliations:** 1 Division of Plant Pathology, ICAR, Indian Agricultural Research Institute, New Delhi 110012, India; 2 Department of Plant Pathology, College of Horticulture, University of Horticultural Sciences, UHS Campus, Bagalkot, GKVK Post, Bengaluru 560065, India; 3 Molecular Biology Lab, ICAR-NBAIM, Maunath Bhanjan 275103, India; 4 Amity Institute of Biotechnology, Amity University Haryana, Manesar, Gurgaon 122413, India; 5 Division of Agriculture Chemical, Plant Pathology, Indian Agricultural Research Institute, New Delhi 110012, India; 6 Department of Botany, Deen Dayal Upadhayay Gorakhpur University, Gorakhpur 273009, India

**Keywords:** bacterial wilt, biological control, mutagens, nitrous acid, *Ralstonia solanacearum*, tomato

## Abstract

Bacterial wilt disease of tomato (*Solanum lycopersicum* L.), incited by *Ralstonia solanacearum* (Smith), is a serious agricultural problem in India. In this investigation, chemical mutagenic agents (NTG and HNO_2_ treatment) and ultraviolet (UV) irradiation have been used to enhance the antagonistic property of *Bacillus amyloliquefaciens* DSBA-11 against *R. solanacearum* UTT-25 towards an effective management of tomato wilt disease. The investigation established the fact that maximum inhibition to *R. solanacearum* UTT-25 was exerted by the derivative strain MHNO_2_-20 treated with nitrous acid (HNO_2_) and then by the derivative strain MNTG-21 treated with NTG. The exertion was significantly higher than that of the parent *B. amyloliquefaciens* DSBA-11. These two potential derivatives *viz*. MNTG-21, MHNO_2_-20 along with MUV-19, and a wild derivative strain of *B. amyloliquefaciens* i.e.,DSBA-11 were selected for GC/MS analysis. Through this analysis 18 major compounds were detected. Among the compounds thus detected, the compound 3-isobutyl hexahydropyrrolo (1,2), pyrazine-1,4-dione (4.67%) was at maximum proportion in the variant MHNO_2_-20 at higher retention time (RT) of 43.19 s. Bio-efficacy assessment observed a record of minimum intensity (9.28%) in wilt disease and the highest bio-control (88.75%) in derivative strain MHNO_2_-20-treated plants after 30 days of inoculation. The derivative strain MHNO_2_-20, developed by treating *B. amyloliquefaciens* with nitrous acid (HNO_2_), was therefore found to have a higher bio-efficacy to control bacterial wilt disease of tomato under glasshouse conditions than a wild-type strain.

## 1. Introduction

Tomato (*Solanum lycopersicon* L.) is one of the most important solanaceous crops which is prone to bacterial wilt disease caused by *Ralstonia solanacearum* (Smith),a soil borne pathogen, under favorable conditions, Yabuuchi et al. [1]. The pathogen has over 450 hosts distributed among54 botanical families and has inflicted heavy yield losses upon various crops worldwide [2].The rate of occurrence of this disease is from 2–60% [3], and the percentage of loss in the yield is 10–92% in tomato plants in India [4,5]. Several management strategies, such as crop sanitation and crop rotation, applying agrochemicals; and using disease-free seeds are generally often observed to get rid of this deadly soil-borne bacterial disease. Moreover 450 hosts distributed among 54 botanical families, and the infusion of chemicals to manage soil-borne disease, may give way to health-related problems among human beings and may spoil the health of the environment as well due to their hazardous nature [6]. As an effective measure towards controlling this wilt disease, an alternative strategy such as using microbes of the *Bacillus* species which belong to the family Bacillaceae and consist of rod-shaped, aerobic or facultative anaerobic endospores. *Bacillus amyloliquefaciens*, an abundant soil inhabitant, is one such microbe and is found in plant root tissues as an endophyte. Endospores, being committed to protect the plants from desiccation, have long shelf life even under adverse conditions. Hence, it is desirable for an effective biocontrol agent [7,8,9].

Although *B. amyloliquefaciens* is a potential bioagent to control several plant diseases, it always has a scope to improve its bio-efficacy against the pathogen towards the management of plant diseases. Physical and chemical mutagens are very useful in improving the efficacy of the bacterial bio-agent strain. In this context, several methods such as ultraviolet irradiation [10], microwave radiation [11], combined physical and chemical methods [12] or site-directed mutagenesis [13] have been used earlier to improve the desired traits of bacteria to achieve better results in comparison to the parent strain. Physical mutagens including ultraviolet (UV) light exert a mutagenic effect by exciting electrons in molecules. Meenu et al., [14] reported that ultraviolet light was the best mutagenic agent. The UV-derivative strains of *Bacillus* have reportedly showed better ability to produce alpha-amylase [15]. However, chemical mutagens are strong mutagenic agents, which compel permanent changes in DNA sequences. Further, they bring about transitions from G:C→A:T [16] and have a preferential effect on the DNA replication [17,18]. Conversion of the amino groups to keto groups changes the hydrogen-bonding potential of the bases. At the same time, nitrous acid has been reported to be a suitable mutagen for the improvement of α-amylase production in *Bacillus* spp. [13,19]. These techniques are often used in *Bacillus* spp. such as *B. subtilis* and *B. amyloliquefaciens*, which are the most commonly, used organisms of choice for amylase and antimicrobial metabolite production [20].

Processes such as survey, rhizospheric soil sample collection, isolation screening of biocontrol activity and plant growth promoting traits are time consuming and cumbersome in characterizing a microbe into a potential biocontrol agent. Under such conditions, the best alternative to this is to develop a derivative strain of a well-established bio-control agent using mutagens. In the present study, physical(UV irradiation) and chemical (NTG (N-methyl-N-nitro-N-nitroso guanidine) and nitrous acid) mutagens have been used to develop potential derivative strains to improve bio-efficacy of *B. amyloliquefaciens* strain DABS-11 against bacterial wilt pathogen *R. solanacearum* and its effect on the production of secondary metabolites by the derivative strains.

## 2. Material and Methods

### 2.1. Bacillus amyloliquefaciens Strain

The *Bacillus amyloliquefaciens* DSBA-11 (NAIMCC-B-1836; antagonistic strain NCBI, accession no: KF850150) and *R. solanacearum* UTT-25 ((NAIMCC-B-1836; pathogenic strain, NCBI accession no: JM113816) were obtained from Plant Bacteriology Laboratory, Division of Plant Pathology, ICAR—Indian Agricultural Research Institute, New Delhi. These cultures were maintained on casein peptone glucose agar (CPG) medium for *R. solanacearum*UTT-25 and TSA medium for *B. amyloliquefaciens* for further studies [7].

### 2.2. Development of Derivative Strains of B. amyloliquefaciens

#### 2.2.1. UV Irradiation Treatment

The mutagenesis of *B. amyloliquefaciens* DSBA- 11 was performed using UV irradiation as described by Radha Krishnan et al. [10]. The bacterial culture was grown in the Luria Bertani (LB) broth medium for 48 h, centrifuged at 5000 rpm for 15 min and washed with 0.9% saline solution (10^8^ cfu/mL). The ultraviolet irradiation was performed using a UV tube (15 W, 2537 A) at 320 nm and the bacterial cell suspension was distributed in 9 cm autoclaved Petri plates (5 mL in each plate), kept at the distance of 30 cm from the UV source and then exposed to UV radiation for a period of 10, 20 and 30 min intermittently. After exposure, 100 µL of exposed sample was taken from each treatment and inoculated on to the LB-medium containing Petri plates and incubated at 37 °C for 72 h. Individual colonies of variant strain were selected for the screening of an inhibition zone against *R. solanacearum*.

#### 2.2.2. Chemical Treatment

Forty-eight-hour-old culture containing 10^8^ cfu/mL of *B. amyloliquefaciens* DSBA-11 was used to develop chemical-based mutagenesis as described by Radha Krishnan et al. [10]. Chemical mutagens i.e., NTG (N-methyl-N-nitro-N-nitroso guanidine) and nitrous acid (HNO_2_) at 50, 100, 150, 200, 250 and 500 µg/mL concentrations were added separately into 5.0 mL of liquid culture of *B. amyloliquefaciens* DSBA-11 and incubated at 37 °C for 30 min on a rotary shaker at 150 rpm in an Orbital shaker incubator (Model-LOM-560, Make-MRC Laboratory Instruments, Jena, Germany). The incubated bacterial mixtures were centrifuged at 5000 rpm for 15 min; we then discarded the supernatant, and washed twice with the phosphate buffer (pH 6.0).The pellet was then suspended into 10.0 mL of sterile distilled water, re-centrifuged thrice on the same conditions, washed carefully and added with 10.0 mL of saline water (0.85% of NaCl). A 100 µL sample of treated bacterial suspension of each treatment was spread on Luria Bertani (LB) agar medium containing Petri plates in triplicate and incubated at 37 °C for 72 h in the Orbital shaker incubator. The death rate of physical as well as chemical mutagenesis was calculated by the formula I% = [1 − (W_t_/W_0_)] ∗ 100% where, I represents death rate, W_t_ represents the number of colonies in the UV irradiation group and W_0_ represents the number of colonies in the blank group [10].

### 2.3. Antagonistic Ability of Developed Derivative Strains of B. amyloliquefaciens against R. solanacearum

To test antagonistic ability of the developed derivative strains of *B. amyloliquefaciens* DSBA-11 against *R. solanacearum* under in vitro conditions as described by Singh et al. [9], the dual culture method was used. The selected derivative strain colonies were grown in Luria broth medium for 4 h at 37 °C and maintained the population of bacteria (0.1 OD at 600 nm). A 100 μL of 48 h old liquid culture (6.2 × 10^8^ cfu/mL) of *R. solanacearum* UTT-25 was spread onto the Petri plates containing the casein peptone glucose agar (CPG) medium separately. Three wells of the diameter 0.5 cm in each Petri plate was made by sterilized cork borer. About 30 μL of derivative strains and wild strain DSBA-11of *B. amyloliquefaciens* were poured in each well separately with three replications. The Petri plates were incubated at 37 °C for 48 h and inhibition zone was recorded. The value of the inhibition zone was converted into an area of inhibition zone using the formula: Area of inhibition zone = πr^2^ [9].

### 2.4. GC/MS Analysis

The three potential antagonistic derivative strains i.e., MUV-19, MNTG-21, MHNO_2_-20 of *B. amyloliquefaciens* along with wild strain *B. amyloliquefaciens* DSBA-11 were selected for GC/MS analysis. One liter of 48 h old culture of these strains grown in Luria broth medium was fractioned with the ethyl acetate (500 mL × 3) separately. The ethyl acetate layer was dried over the anhydrous sodium sulphate and under reduced pressure at 45 °C it was collected and dried. Metabolite profiles of the ethyl acetate extract were determined by using gas chromatography and mass spectrometry (Focus-DSQ, Thermo Scientifc, New Delhi, India) equipped with a DB-5 capillary column of the size of 30 m × 0.25 mm and thefilm of thickness of 0.25 µm. Conditions observed while using gas chromatography were: 1. helium was used as the carrier gas with the flow rate of 1 mL/min (split mode, 1:20); injection with the volume 1.0 µL (10 mg extract/3 mL acetone); and column temperature maintained at 60 °C and then programmed at 3 °C/min to 280 °C for 5 min. The injector ion source and mass spectrometric transfer line temperatures were kept at 250, 230 and 280 °C. The column was coupled directly to a quadruple mass spectrometer (EI mode, at 70 eV) with the mass range 28–500 a.m.u at 1 scan/s. The compounds were individually identified by comparing their mass spectrum with the spectrum of the compound available in the NIST Mass Spectral Library and literature [21].

### 2.5. Motility of Derivative and Wild Strains of B. amyloliquefaciens

Luria Bertoni agar (LA) and nutrient agar (NA) media containing 1.0 and 1.5 % concentration of agar powder, respectively, with three replications were used to study the motility rate in terms of colony growth of derivative strains MUV-19, MNTG-21, MHNO_2_-20 and wild strain DSBA-11 at 24 h intervals up to 96 h. The fresh colonies of these strains were then placed at the center of the Petri plate and incubated at 30 °C for 96 h. The diameter (in centimeters) of the bacterial colony, thus established, was measured at 24 h intervals.

### 2.6. Bacterial Wilt Disease Control of Tomato by Using Derivative and Wild Strains of B. amyloliquefaciens

The three best potential derivative strains *viz.*, MUV-19, MNTG-21, MHNO_2_-20 and the wild strain DSBA-11 of *B. amyloliquefaciens* were used to study the bio-efficacy against bacterial wilt and plant-growth-promoting activities in tomato cv. Pusa Ruby under controlled conditions provided by the National Phytotron Facility, IARI, New Delhi, India. Twenty-one-day-old seedlings of tomato cv. Pusa Ruby were transplanted in the pots of 15.0 cm diameter which contained an autoclaved soil mixture of peat moss, vermiculite and sand in the ratio 2:1:1 at 25–30 °C, as well as 48 h old colonies of *R. solanacearum* and derivative strains along with wild strain DSBA- 11 of *B. amyloliquefaciens* containing bacterial population of 0.1 OD at 600 nm measured by spectrophotometer. After five days of transplanting five seedlings into each pot with three replications, 5.0 mL of liquid culture of *R. solanacearum* UTT-25 was inoculated at the root zone of the plants. Subsequently, the same amount of derivative strains and wild strain DSBA-11were inoculated at the root zone of the plants separately. The plants treated with *R. solanacearum* only, and sterilized distilled water-inoculated plants were also maintained to serve as a positive and negative control with three replications, respectively. The observations were recorded at sevendays of intervals up to 28 days after inoculation. The percentage (1–100%) for wilt disease intensity was recorded at the initial stage and final stage (the whole plant wilted). Disease rating was also recorded by using a1–5 scale and wilt intensity was measured as described by Schaad et al. [22]. The biological control efficacy (BCE) of antagonistic bacteria was determined as described by Guo et al. [23].The whole plants with roots were uprooted from each treatment with three replicates. The root and shoot of each plant were cut from the crown region for length (cm) measurement, and fresh weight and dry weight (60 °C for 3 days) were taken. The growth promotion efficacy (GPE) of *B. amyloliquefaciens* based on plant dry weight was calculated as described by Singh et al. [9].

### 2.7. Statistical Analysis

The analysis of the variance for the biocontrol efficiency and yield of tomato was completed by the SAS general linear model (GLM) procedure (SAS Institute, Version 6, Cary, NC, USA). The mean comparison was conducted by the least significant difference (LSD) test (*p* = 0.05). Standard error and LSD results were recorded.

## 3. Results

### 3.1. Development of Variant Strains and Their Antagonistic Activity against R. solanacearum under In Vitro Conditions

The *B. amyloliquefaciens* DSBA-11 was exposed to UV irradiation and the observed lethality of derivative strain was increased by 53.11 to 85.75% with increase in the exposure time period from 10–30 min. A total of 94 derivative colonies were screened for antagonistic ability, though only 20 derivative colonies (21.27%) showed antagonistic potential against *R. solanacearum* under in vitro conditions. Moreover, the derivative strain MUV-19 (30 min exposure) showed the highest inhibition zone (3.42 cm^2^) against *R. solanacearum* UTT-25, which was slightly higher than wild strain DSBA-11 (3.35 cm^2^), while the remaining derivative strains obtained from UV irradiation showed a smaller inhibition zone against *R. solanacearum* UTT-25 than wild strain DSBA-11 (Table 1).

*B. amyloliquefaciens* DSBA-11 culture was treated with chemical mutagens i.e., NTG (N-methyl-N-nitro-N-nitroso guanidine) and HNO_2_ at concentrations of 50, 100, 150, 200, 250 and 500 µg/mL and lethality rate was found comparatively higher in HNO_2_-treated bacterial cells (91.4 and 95.7%) than NTG-treated cells (85.88 and 90.32%) at 250 and 500 µg/mL concentrations respectively (Table 2). It was found that the increase in the concentration of chemical mutagens increased the lethality rate of *B. amyloliquefaciens* DSBA-11. Out of 71 survived derivative strain colonies chosen from NGT treatment, only 23 derivative strains (32.34%) had antagonistic ability against *R. solanacearum* UTT-25 to form an inhibition zone. In contrast, five derivative strains *viz.* MNTG-17, MNTG-18, MNTG-20, MNTG-21 and MNTG-22, obtained at 250 and 500 µg/mL concentrations, formed an inhibition zone ranging from 3.40 to 3.56 cm^2^ against *R. solanacearum* UTT-25. They showed comparatively more antagonistic ability to form an inhibition zone than wild strain DSBA-11 (3.35 cm^2^) (Table 2). In HNO_2_-treated *B*. *amyloliquefaciens* DSBA-11 cells, a total of 52 survived derivative strain colonies were taken to test their antagonistic activity against *R. solanacearum* UTT-25, but only 22.98% derivative strain colonies showed antagonistic ability to form an inhibition zone against *R. solanacearum*. However, four derivative strains i.e., MHNO_2_-16, MHNO_2_-18, MHNO_2_-19 and MHNO_2_-20, showed comparatively higher antagonistic activity to form an inhibition zone ranging from 3.70–4.60 cm^2^ against *R. solanacearum* UTT-25 than wild strain DSBA-11 (3.35 cm^2^) under in vitro conditions (Table 2).

### 3.2. GC/MS Analysis

**Figure 1:**
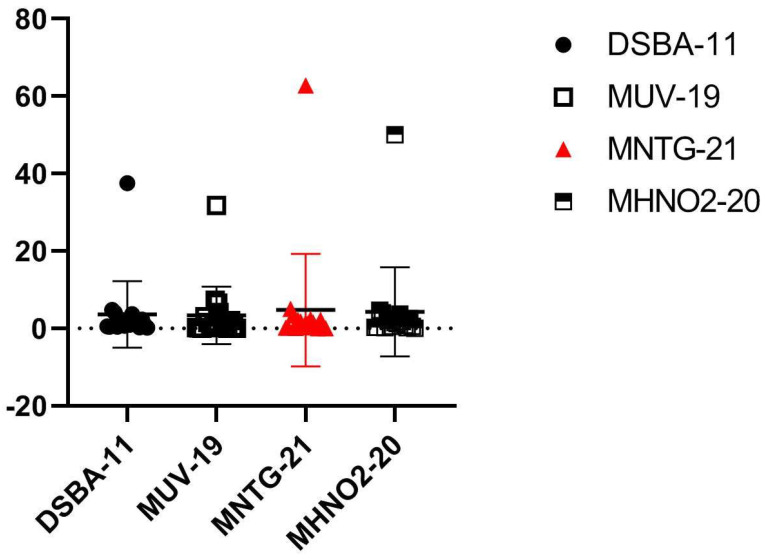
Scattered plot graph showing gas chromatography–mass spectrometry (GC–MS) area profile of major volatile organic compounds (VOCs) produced by derivative strains and parent strain of *B. amyloliquefaciens* DSBA-11 under in vitro conditions.

**Figure 2:**
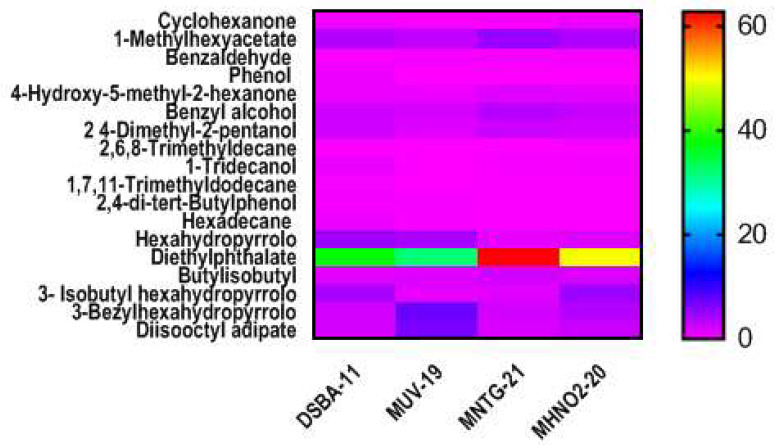
Heatmap showing major chemical compounds identified in the ethyl acetate extract of the derivative strain and wild strain of *B. amyloliquefaciens* DSBA-11.

In ethyl acetate extract, 18 major compounds were identified from the three derivative strains MNTG-21, MUV-19, MHNO_2_-20, and wild DSBA-11. The major compounds thus found were hexahydropyrrolo (1,2-a)pyrazine-1,4-dione; diethylphthalate; butylisobutyl phthalate; 3-Isobutylhexahydropyrrolo (1,2-a)pyrazine-1,4-dione; 3-bezyl-hexahydro-pyrrolo(1,2-a) pyrazine-1,4-dione; and diisooctyladipate, besides other compounds in minor amounts (Table 3). The derivative strainMHNO_2_-20-treated with HNO_2_ produced the maximum amount of compounds such as3- isobutylhexahydropyrrolo (1,2-a)pyrazine-1,4-dione (4.67%); and 3-bezyl-hexahydro-pyrrolo(1,2-a) pyrazine-1,4-dione(3.73%) at retention times of 43.19 and 56.34 s, respectively. On the other hand, diethylphthalate (62.82%) was identified in the NTG-treated strain MNTG-21. The variations in production of different metabolites by the derivative strains of *B. amyloliquefaciens* along with the parent strain and their quantities were recorded (Table 3, Figure 1). The heat map showed major chemical compounds identified in the ethyl acetate extract of the derivative strain and wild strain DSBA-11 (Figure 2).

### 3.3. Motility of Derivative and Wild Strains

**Figure 3:**
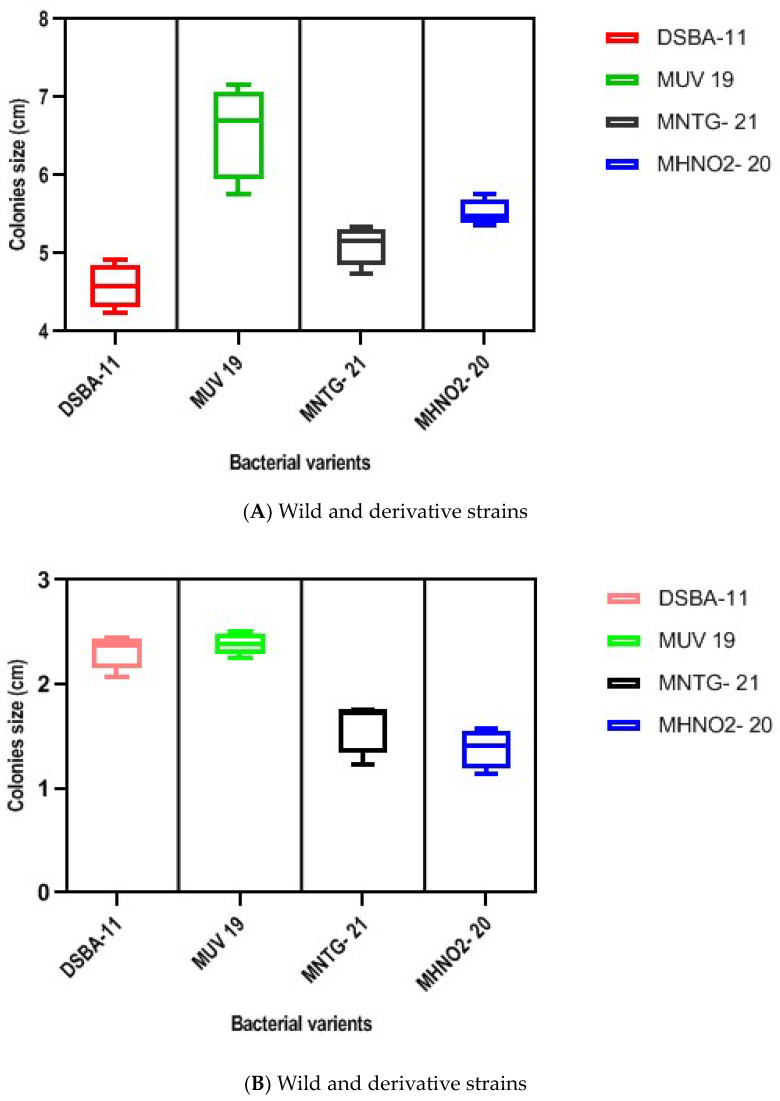
Motility of *B. amyloliquefaciens* DSBA-11 and its derivative strains on nutrient agar medium at 1.0% (**A**) and 1.5% (**B**) concentrations.

**Figure 4:**
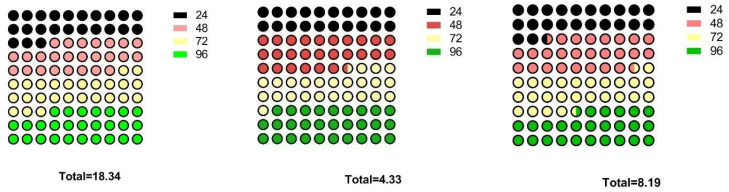
Dot box pictorial representation for the motility of *B. amyloliquefaciens* DSBA-11 and its derivative strains at different time intervals.

Three derivative strains *viz.*, MUV-19, MNTG-21, MHNO_2_-20 and wild strain DSBA-11 were streaked on nutrient agar and LA-media containing 1.0 and 1.5% agar and allowed to grow for 96 h. The derivative strains showed faster growth than wild strain DSBA-11 on both the solid media. Composition of medium and agar powder concentrations affected the mobility of the bacteria significantly. LA-medium supported better growth of wild strain DSBA-11 and derivative strains than NA medium. However, LA medium @ 1.0% conc. of agar was found to be the best-suited medium for the growth of derivative strains as well as wild strain DSBA-11 after 96 h of incubation. The maximum colony diameter was found in MUV-19 (7.15 cm) followed by MHNO_2_-20 (5.76 cm) and MNTG-21 (5.35 cm), which were significantly higher than for wild strain DSBA-11 (4.92 cm) after 96 h of incubation (Table 4).The box plot represents the motility of *B. amyloliquefaciens* DSBA-11 and its derivative strains on different media at different concentrations of agar powder (Figure 3A,B and Figure 4).

### 3.4. Biocontrol Efficacy and Plant Growth-Promoting Activity of Derivative Strains of B. amyloliquefaciens DSBA-11

**Figure 5:**
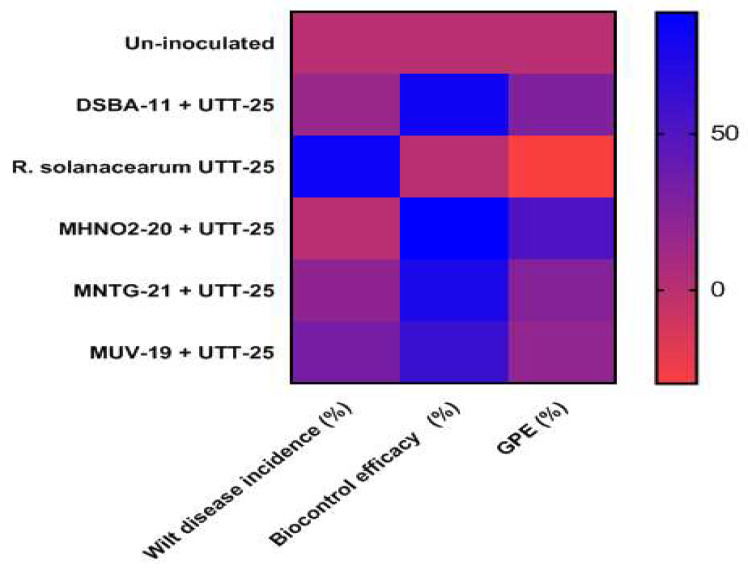
Heat map showing indirect proportionality relationship between wilt incidence and biocontrol efficacy by various treatments of *B. amyloliquefaciens* DSBA-11 and its derivative strains for enhancement in biomass of tomato cv. Pusa Ruby.

The derivative strains of *B. amyloliquefaciens* DSBA-11, MUV-19, MNTG-21 and MHNO_2_-20 reduced the intensity of bacterial wilt disease under controlled conditions at the Phytotron facility, IARI, New Delhi. Minimum wilt disease intensity (9.28%) was observed with the highest biocontrol efficacy (88.75%) in MHNO_2_-20-treated tomato cv. Pusa Ruby plants followed by DSBA-11-treated plants (15.40%) after 30 days of the inoculation of the pathogen, which was significantly lower than for *R. solanacearum*-treated plants (82.57%). Other derivative strains such as MUV-19 and MNTG-21 had a biocontrol efficiency of 75.47 and 60.59%, respectively, and showed lower incidence of wilt in tomato plants than solely *R. solanacearum*-treated plants. The vigorous growth of tomato cv. Pusa Ruby was recorded with a maximum root (7.09 cm) and shoots (67.86 cm) length in MHNO_2_-20-treated plants followed by DSBA-11 and MNTG-21-treated plants. The biomass of the dry weight of plants treated by the derivative strains MHNO_2_-20 were recorded to be highest with GPE 51.79% followed by the parent strain DSBA-11 (27.88%) and MNTG-21 (24.70%). However, only *R. solanacearum*-treated plants showed comparatively lesser growth and total biomass (GPE: −29.88%) than untreated control (Table 5, Figure 5).

## 4. Discussion

Bacterial wilt caused by *R. solanacearum* is a serious threat to tomato crops in disease-prone areas across the world due to its soil-borne nature and wide host range [10,24]. Management of bacterial wilt disease with microbes, including antagonistic fungi and bacteria, is an alternative technique that is safe, environmentally benign, less expensive, and more sustainable [7,25,26]. In this investigation, we employed *B. amyloliquefaciens* DSBA-11, a possible antagonistic bacterium used to manage tomato bacterial wilt disease [7,27] to improve its antagonistic capacity and growth boosting activity. The UV irradiation (15 W, 2537 A at 320 nm for 10–30 min of exposure duration) and chemical mutagens such as NTG and HNO_2_ at concentrations of 50–500 µg/mL were employed to create derivative strains in mutagenesis bacteria [28]. In *Pseudomonas fluorescens* [10] and *Bacillus* sp. utilized a similar strategy of UV irradiation for the production of derivative strains, whereas Szafraniec et al. [19] and Karanam et al. [13] in *Bacillus* sp. used chemical mutagens. In the present study, the lethality rate of *B. amyloliquefaciens* was found to be the highest at 500 µg/mL of both the chemical mutagens i.e., NTG (90.32%) and nitrous acid (95.70%) as compared to UV irradiation (85.75%) at 30 min of exposure. However, the concentration of chemical mutagens and also the exposure duration in UV irradiation decreased the lethality rate. The best three derivative strains, MUV-19 (UV-treated), MNTG-21, and MHNO2-20, demonstrated the strongest antagonistic activity against *R. solanacearum* in each category of mutagenic agents. However, the maximum inhibition zone of 4.6 cm^2^ was formed by MHNO_2_-20 (obtained from nitrous acid treatment at 500 µg/mL) followed by, 3.56 cm^2^ by MNTG-21 (NTG-treated) and 3.42 cm^2^ by the strain MUV-19 (30 min exposure time), which was significantly higher than the wild strain DSBA-11 (3.35 cm^2^). The outcomes showed that the bioefficacy of the *B. amyloliquefaciens* developed derivative strains against *R. solanacearum* had significantly increased [28], where they used UV light to improve the antibiosis of *P. fluorescens* (including phenazine, pyrrolnitrin, and phloroglucinol) and siderophores production against damping-off pathogens (*Fusarium solani*, *F. oxysporum*, and *Rhizoctonia solani*). The improvement of actinomycete bacterial strains by using gamma irradiation for enhancing chitinase production activity by induced derivative strain was also reported by Rugthaworn et al. [29] and was found to exhibit a higher inhibitory effect on *F. sporotrichioides*, *R. solani*, and *Sclerotium rolfsii*. Additionally, we employed the wild strain DSBA-11 and three of the most promising derivative strains, MUV-19, MNTG-21, and MHNO_2_-20, in this study to examine the bioefficacy against the bacterial wilt disease in tomato cv. Pusa Ruby under controlled conditions. The results showed wilt intensity in tomato declined significantly (*p* < 0.05) in MHNO_2_-20-treated plants (Table 5) with the best biocontrol efficacy (88.75) among other treatments. The derivative strain MHNO_2_-20 also promoted plant growth by showing the best growth efficacy (51.79%) followed by wild strain DSBA-11 (27.88%).

We also observed that among the three different mutagens used for development of derivative strains of *B. amyloliquefaciens* DSBA-11, the derivative MHNO_2_-20 showed significantly highest bioefficacy to inhibit the growth of *R. solanacearum* and similar result was reported by Haq et al. [30], in which, they found that nitrous acid was found to be the suitable mutagen for improvement of *Bacillus* spp. Similarly, Sarikaya [11], Haq [31] and Varalakshmi et al. [32] reported that the UV irradiation-treated *Bacillus* spp. was found most effective in the production of α-amylase. In this study, we observed that the derivative strains developed at lower concentration (50–100 µg/mL) of NGT and nitrous acid and short duration of exposure to UV irradiation showed lesser bioefficacy than wild DSBA-11 (Table 1 and Table 2). The similar decreasing trend was also reported in the production of α-amylase in a mutant strain of the *B. amyloliquefaciens*, UNG-16, when compared to the wild strain [33]. Moreover, as per the records of our study, the chance of isolating an effective derivative with the desired trait is lower, in the sense that it was approximately 0.11, 7.0 and 7.4%. Developed derivative strains using UV irradiation, NGT and nitrous acid treatment showed better bioefficacy than wild strain DSBA-11 to form an inhibition zone against *R. solanacearum* under in vitro conditions. However, under in vivo conditions, out of the three derivative strains used, only the strain MHNO_2_-20 showed a better performance in biocontrol efficacy and plant growth efficacy than the wild strain DSBA-11 (Table 5).

*Bacillus* spp. secretes various secondary metabolites (including antibiotics, antifungals and siderophores) which can affect the microbiota in the rhizosphere providing an environment antagonistic to pathogens, or trigger host defense responses [34]. In this investigation, a comparative analysis of secondary metabolites produced by derivative strains and wild strain DSBA-11 was analyzed by using GC/MS. Although we found variation in the production of volatile compounds by derivative strains *viz.*, MUV-19, MNTG-21, MHNO_2_-20 and DSBA-11 as mentioned in Table 3, the derivative strain MHNO_2_-20 produced the highest quantity of 3-isobutyl hexahydropyrrolo (1,2-a) pyrazine-1,4-dione and 3-bezylhexahydropyrrolo (1,2-a) pyrazine-1,4-dione while derivative strains MNTG-21 produced diethylphthalate. However, these compounds could not validate against *R. solanacearum*, though they open the scope of the metabolomics approach in host pathogen interaction studies. *Bacillus* spp. have ability to extensively colonize the surface of semi-solid media by a flagellum independent mechanism and reported that sliding motility is responsible for surface migration [35]. Moreover, the surface colonization is also dependent on the secretion of surfactin, but microscopic examination of the edges and interior cells of sliding surface colonies did not reveal abundant flagella. Fall et al. [36] suggested that *B. subtilis* has two distinct modes of surface translocation *viz.*, swarming and sliding, which are presumably advantageous under different environmental conditions. In the present study, we observed that motility was comparatively higher in all derivative strains i.e., MUV-19, MHNO_2_-20 and MNTG-21, than in the wild strain DSBA-11 after 96 h (Table 4). The results showed that the spreading behavior of *B. amyloliquefaciens* was enhanced on the semi-solid media after bringing it under UV irradiation and then treating with the chemical mutagens. The essential macro- and micronutrients were needed for the sliding motility and the colony spreading as reported by Fall et al. [36] and it was confirmatory to our work. We found that the diameter of the colony of both wild strain and derivative strains was larger on the LA medium as compared to the NA medium after 96 h of incubation at 30 °C (Figure 5). Further, we found that the level of hardness of the surface of the medium also affected the spreading nature of the bacteria. A 1.0% conc. of agar powder in NA and LA media was found better for the growth of variant strain and parent strain DSBA-11 than a 1.5% conc. of agar because it might have supported swarm or twitching motility of the bacteria on the soft surface of the media in *B. subtilis* as reported earlier by Fall et al. [36] and in *Pseudomonas aeruginosa* as reported by Rashid et al. [37]. Ho et al. [38] used the *B. amyloliquefaciens* strain PMB05 for bacterial wilt disease management, and observed the increase in the signals of Pop W-induced reactive oxygen species generation and callose deposition and thereby confirmed that the PTI was intensified by PMB05.

## 5. Conclusions

The following conclusions have been drawn from the results of the study:

➢ In comparison to variant strains created by NTG and UV irradiation, the variant strains MHNO2-20 of *B. amyloliquefaciens* andDSBA-11 were reported to have greater antagonistic potential against *R. pseudosolanacearum.*
➢ It was also observed that, using nitrous acid at 250–500 µg/mL, it was concluded that a high rate of lethality of bacteria treated with chemical and physical mutagens developed a better chance to obtain a potential variant strain with desired traits.
➢ Under in vitro circumstances the nitrous acid-treated variant strains were successful particularly in controlling bacteria wilt disease.
➢ Additionally, it was observed that chemical mutagens were more efficient than physical mutagens in obtaining the necessary variant strain of *B. amyloliquefaciens* with better antagonistic ability.

## Figures and Tables

**Table 1:** Lethality rate and antagonistic activity of derivative and wild strains of *B. amyloliquefaciens* against *R. solanacearum* developed by ultraviolet irradiation under in vitro conditions.

Duration of Exposure (Min)	Lethality Rate (%)	Variant Strain	Area of Inhibition Zone (cm^2^)
		MUV-5	1.13 ^ij^
10	53.11	MUV-8	1.80 ^g^
		MUV-9	1.73 ^g^
		MUV-10	1.66 ^g^
20	57.4	MUV-11	2.56 ^de^
		MUV-15	2.40 ^ef^
		MUV-14	2.23 ^f^
30	85.75	MUV-16	3.10 ^bc^
		MUV-19	3.42 ^ab^
		MUV-20	3.23 ^ab^
-	-	DSBA-11 *	3.35 ^a^

* Wild strain of *B. amyloliquefaciens*. The mean comparison was conducted by the least significant difference (LSD) test (*p* = 0.05). Different letters point out significant differences in a column. Data present means of the experiment within 3 replications each.

**Table 2:** Lethality rate and antagonistic activity of derivative and wild strains of *B. amyloliquefaciens* against *R. solanacearum* developed by using nitroso guanidine (NTG) and nitrous acid (HNO_2_) under in vitro conditions.

NTG Treatment				HNO_2_ Treatment		
Concentration of Chemical Mutagen (μg/mL)	Lethality Rate (%)	Isolate Code	Area of Inhibition Zone (cm^2^)	Lethality Rate (%)	Isolate Code	Area of Inhibition Zone (cm^2^)
50	61.02	MNTG-1	2.70 ^defg^	58.59	MHNO_2_-2	1.10 ^ij^
		MNTG-2	2.10 ^fghij^		MHNO_2_-3	1.06 ^j^
100	69.22	MNTG-6	1.70 ^hij^	66.27	MHNO_2_-5	1.33 ^hij^
		MNTG-7	1.66 ^ij^		MHNO_2_-6	1.40 ^hi^
150	74.74	MNTG-9	2.06 ^ghij^	80.6	MHNO_2_-7	1.70 ^fg^
		MNTG-10	2.46 ^fghi^		MHNO_2_-8	1.56 ^fgh^
		MNTG-11	2.73 ^cdefg^		MHNO_2_-9	1.26 ^hij^
200	80.91	MNTG-13	2.83 ^bcdef^	88.15	MHNO_2_-10	1.83 ^f^
		MNTG-14	2.93 ^bcdef^		MHNO_2_-11	1.83 ^f^
		MNTG-15	3.30 ^abcde^		MHNO_2_-11	2.84 ^e^
250	85.88	MNTG-16	3.33 ^abcd^	91.40	MHNO_2_-13	2.46 ^e^
		MNTG-17	3.40 ^abcd^		MHNO_2_-14	2.63 ^e^
		MNTG-18	3.50 ^abcd^		MHNO_2_-16	3.70 ^c^
500	90.32	MNTG-20	3.43 ^abcd^	95.70	MHNO_2_-18	4.50 ^a^
		MNTG-21	3.56 ^abc^		MHNO_2_-19	4.40 ^b^
		MNTG-22	3.46 ^ab^		MHNO_2_-20	4.60 ^a^
	-	DSBA-11 *	3.35 ^a^	-	DSBA-11 *	3.35 ^d^

* Parent strain of *B. amyloliquefaciens*. The mean comparison was conducted by the least significant difference (LSD) test (*p* = 0.05). Different letters point out significant differences in a column. Data present means of the experiment within 3 replications each.

**Table 3:** Chemical compounds identified in the ethyl acetate extract of the derivative and wild strains of *B. amyloliquefaciens* DSBA-11.

Compounds	DSBA-11	MUV-19	MNTG-21	MHNO_2_-20	RT
Cyclohexanone	0.58	0.22	0.11	0.79	4.75
1-Methylhexyacetate	3.68	3.05	5.01	4.01	5.23
Benzaldehyde	0.32	0.40	0.22	0.58	6.42
Phenol	0.73	-	-	-	7.01
4-Hydroxy-5-methyl-2-hexanone	0.89	0.83	1.48	1.33	7.17
Benzyl alcohol	2.35	2.03	3.39	2.66	7.89
2,4-Dimethyl-2-pentanol	2.27	1.54	2.37	2.13	14.95
2,6,8-Trimethyldecane	0.22	0.07	0.07	0.21	15.39
1-Tridecanol	0.96	0.09	0.37	0.54	23.55
2,7,11-Trimethyldodecane	0.43	0.04	0.29	0.28	23.89
2,4-di-tert-Butylphenol	0.47	0.34	0.18	0.18	28.47
Hexadecane	0.9	0.23	0.27	0.27	31.55
Hexahydropyrrolo (1,2,α) pyrazine-1,4-dione	4.78	4.10	1.27	1.57	39.86
Diethylphthalate	37.55	31.80	62.82	50.0	41.38
Butylisobutyl phthalate	1.31	1.58	2.23	1.82	42.92
3- Isobutyl hexahydropyrrolo (1,2,α) pyrazine-1,4-dione	4.13	1.26	1.56	4.67	43.19
3-Bezylhexahydropyrrolo (1,2,α) pyrazine-1,4-dione	2.16	7.26	2.22	3.73	56.34
Diisooctyl adipate	1.95	6.55	1.78	2.57	57.32

**Table 4:** Motility of *B. amyloliquefaciens* DSBA-11 and its derivative strains on the different media and agar powder concentrations.

Media and Concentration (%)	Duration (h)	Colony Size of Bacteria (Diameter in cm)			
		DSBA-11	MUV 19	MNTG-21	MHNO_2_-20
Luria agar (1%)	24	4.24 ^d^	5.75 ^d^	4.74 ^c^	5.36 ^d^
	48	4.55 ^c^	6.56 ^c^	5.15 ^b^	5.45 ^c^
	72	4.63 ^b^	6.85 ^b^	5.16 ^b^	5.5 ^b^
	96	4.92 ^a^	7.15 ^a^	5.35 ^a^	5.76 ^a^
Luria agar (1.5%)	24	0.86 ^j^	1.27 ^j^	1.35 ^j^	1.15 ^m^
	48	1.15 ^i^	1.37 ^ij^	1.47 ^i^	1.26 ^l^
	72	1.07 ^i^	1.37 ^ij^	1.43 ^i^	1.26 ^l^
	96	1.25 ^h^	1.46 ^i^	1.7 ^h^	1.36 ^k^
Nutrient agar (1%)	24	1.86 ^g^	4.2 ^f^	1.8 ^fg^	2.16 ^h^
	48	2.13 ^f^	4.3 ^f^	1.85 ^f^	2.26 ^g^
	72	2.13 ^f^	4.5 ^e^	2.13 ^d^	2.4 ^f^
	96	2.07 ^f^	4.66 ^e^	2.03 ^e^	2.56 ^e^
Nutrient agar (1.5%)	24	2.07 ^f^	2.25 ^h^	1.23 ^k^	1.14 ^m^
	48	2.37 ^e^	2.37 ^h^	1.7 ^h^	1.36 ^k^
	72	2.37 ^e^	2.4 ^h^	1.75 ^gh^	1.45 ^j^
	96	2.45 ^e^	2.5 ^g^	1.76 ^fgh^	1.58 ^i^

The mean comparison was conducted by the least significant difference (LSD) test (*p* = 0.05). Different letters point out significant differences in a column. Data present means of the experiment within 3 replications each.

**Table 5:** Reduction of bacterial wilt disease and enhancement of biomass of tomato cv. Pusa Ruby treated with *B. amyloliquefaciens* DSBA-11 and its derivative strains.

Treatments	Wilt Disease Incidence (%)	Biocontrol Efficacy (%)	Length of Plant (cm)		Dry Weight (gm/Plant)		GPE (%)
			Root	Shoot	Root	Shoot	
Un-inoculated	0 ^f*^	-	3.16 ^e^	31.96 ^e^	0.62 ^e^	1.89 ^e^	-
DSBA-11 + UTT-25	15.40 ^d^	81.35	6.16 ^b^	56.10 ^b^	0.95 ^b^	2.26 ^b^	27.88
*R. solanacearum* UTT-25	82.57 ^a^	-	2.92 ^f^	28.36 ^f^	0.49 ^f^	1.27 ^f^	−29.88
MHNO_2_-20 + UTT-25	9.28 ^e^	88.75	7.09 ^a^	67.85 ^a^	1.03 ^a^	2.78 ^a^	51.79
MNTG-21 + UTT-25	20.28 ^c^	75.47	4.33 ^c^	51.40 ^c^	0.98 ^c^	2.15 ^c^	24.70
MUV-19 + UTT-25	32.55 ^b^	60.59	3.66 ^d^	39.70 ^d^	0.91 ^d^	2.06 ^d^	18.81

The mean comparison was conducted by the least significant difference (LSD) test (*p* = 0.05). * Different letters point out significant differences in a column. Data present means of the experiment within 3 replications each.

## Data Availability

Not applicable.

## References

[1] Yabuuchi E., Kosako Y., Yano I., Hotta I., Nishiucliy Y. (1995). Transfer of two *Burkholderia* and an *Alcaligenes* species to *Ralstonia* General Nov: Proposal of *Ralstoniapickettii* (*Ralston*, palleroni and Doudoroff 1973) Comb. Nov, *Ralstonia solanacearum* (Smith1896) Comb. Nov. Microbiol. Immunol..

[2] Sharma J.P., Kumar S., Singh D. (2015). Bacterial Wilt of Solanaceous Crops.

[3] Singh D., Sinha S., Yadav D.K., Sharma J.P., Srivastava D.K., Lal H.C., Mondal K.K., Jaiswal R.K. (2010). Characterization of biovar/races of *Ralstonia solanacearum*, the incitant of bacterial wilt in solanaceous crops. Indian Phytopath..

[4] Mishra A., Mishra S.K., Karmakar S.K., Sarangi C.R., Sahu G.S. (1995). Assessment of yield loss due to wilting in some popular tomato cultivars. Environ. Ecol..

[5] Artal R.B., Gopalkrishnan C., Thippeswamy B. (2012). An efficient inoculation method to screen tomato, brinjal and chilli entries for bacterial wilt resistance. Pest Manag. Hortic. Ecosyst..

[6] Aslam N., Tariq M., Hussain M.A., Raheel M. (2017). Assessment of resistance to bacterial wilt incited by *Ralstonia solanacearum* in tomato germplasm. J. Plant Dis. Prot..

[7] Singh D., Yadav D.K., Chaudhary G., Rana V.S., Sharma R.K. (2016). Potential of *Bacillus amyloliquefaciens* for biocontrol of bacterial wilt of tomato incited by *Ralstonia solanacearum*. J. Plant Pathol. Microbiol..

[8] Lin C., Tsai C.H., Chen P.Y., Wu C.Y., Chang Y.L., Yang Y.L., Chen Y.L. (2018). Biological control of potato common scab by *Bacillus amyloliquefaciens* Ba01. PLoS ONE.

[9] Singh D., Yadav D.K., Sinha S., Upadhyay B.K. (2012). Utilization of plant growth promoting *Bacillus subtilis* isolates for the management of bacterial wilt incidence in tomato caused by *Ralstoniasolanacearum* race 1 biovar3. Indian Phytopath..

[10] Radha Krishnan E., Kumar S.P., Kumar V.B. (2011). Strain improvement of selected strain *Bacillus subtilis* (MTCC No.10619) for enhanced production of antimicrobial metabolites. J. Microbiol. Biotech. Res..

[11] Sarikaya E., Gürgün V. (2000). Increase of the a-amylase yield by some *Bacillus* strains. Turk. J. Biol..

[12] Afifi A.F., Abo-Elmagd H.I., Housseiny M.M. (2014). Improvement of alkaline protease production by *Penicillium chrysogenum* NRRL 792 through physical and chemical mutation, optimization, characterization and genetic variation between mutant and wild-type strains. Ann. Microbiol..

[13] Karanam S.K., Medicherla N.R. (2008). Enhanced lipase production by mutation induced *Aspergillusjaponicus*. Afr. J. Biotechnol..

[14] Meenu M., Santhoshm D., Kamia C., Randhir S. (2000). Strain improvement of *Aspergillus flavus* for enhanced production. Indian J. Microbiol..

[15] Rivera M.H., López-Munguía A., Soberón X., Saab-Rincón G. (2003). Alphaamylase from *Bacillus licheniformis* mutant near to the catalytic site: Effects on hydrolytic and transglycosylation activity. Protein Eng..

[16] Miller J.H. (1972). Experiments in Molecular Genetics.

[17] Wu C.H., Apweiler R., Bairoch A., Natale D.A., Barker W.C. (2006). The universal protein resource (UniProt): An expanding universe of protein information. Nucleic Acids Res..

[18] Besoain X.A., Pérez L.M., Araya A., Lefever L., Sanguinetti M., Montealegre J.R. (2007). New strains obtained after UV treatment and protoplast fusion of native *Trichodermaharzianum*: Their biocontrol activity on *Pyrenochaetalycopersici*. Elec. J. Biotechnol..

[19] Szafraniec K., Wloch D.M., Sliwam P., Bortsm R.H., Koron R. (2003). Small fitness effects and weak genetic interactions between deleterious mutations in heterozygous loci of the yeast *Saccharomyces cerevisiae*. Genet. Res..

[20] Ikram-Ul-Haq S., Saleem A., Javed M.M. (2009). Mutagenesis of *Bacillus licheniformis* through ethyl methane sulfonate for alpha amylase production. Pak. J. Bot..

[21] Adams R.P. (2007). Identification of Essential Oil Components by Gas Chromatography/Mass Spectroscopy.

[22] Schaad N.W., Jones J.B., Chun W. (2001). Laboratory Guide for the Identification of Plant Pathogenic Bacteria.

[23] Guo J., Guo Y., Zhang L., Qi H., Fang Z.D. (2004). Screening for biocontrol agents against *Ralstonia solanacearum*. Chin. J. Biol. Control.

[24] Hayward A.C. (1991). Biology and epidemiology of bacterial wilt caused by *Pseudomonas solanacearum*. Annu. Rev. Phytopathol..

[25] Elsayed T.R., Jacquiod S., Nour E.H., Sørensen S.J., Smalla K. (2020). Biocontrol of bacterial wilt disease through complex interaction between tomato plant, antagonists, the indigenous rhizospheremicrobiota and *Ralstoniasolanacearum*. Front. Microbiol..

[26] Nion Y.A., Toyota K. (2015). Recent trends in control methods for bacterial wilt diseases caused by *Ralstonia solanacearum*. Microbes Environ..

[27] Yadav D.K., Singh D., Kumar N. (2017). Induction of defense-related enzymes by *Bacillus amyloliquefaciens* DSBA-11 in resistant and susceptible cultivars of tomato against bacterial wilt disease. Int. J. Agric. Res..

[28] Haggag W.M. (1999). Enhancement of suppressive metabolites from *Pseudomonas fluorescens* against tomato damping-off pathogens. Arab. J. Biotechnol..

[29] Rugthaworn P., Dilokkunanant U., Sangchote S., Piadang N., Kitpreechavanich V. (2007). A search and improvement of actinomycete strains for biological control of plant pathogens. Kasetsart J. Nat. Sci..

[30] Haq I., Ashraf H., Rani S., Qadeer M.A. (2002). Biosynthesis of alpha amylase by chemically treated mutant of *Bacillus subtilis* GCBU-20. Pak. J. Biol. Sci..

[31] Haq I., Ashraf H., Abdullah R., Shah A.H. (2002). Isolation and screening of Fungi for the biosynthesis of alpha- amylase. Biotechnology.

[32] Varalakshmi K.N., Kumudini B.S., Nandini B.N., Solomon J.D., Mahesh B. (2008). Characterization of Alpha Amylase from *Bacillus* sp. isolated from paddy seeds. J. Appl. Bio. Sci..

[33] Haq I., Ali S., Javed M.M., Hameed U., Saleem A. (2010). Production of alpha amylase from a randomly induced mutant strain of *Bacillus amyloliquefaciens* and its application as a desizer in textile industry. Pak. J. Bot..

[34] Singh D., Yadav D.K., Sinha S., Mondal K.K., Singh G., Pandey R.R., Singh R. (2013). Genetic diversity of iturin producing strains of *Bacillus* species antagonistic to *Ralstonia solanacearum* causing bacterial wilt disease in tomato. Afr. J. Microbiol. Res..

[35] Kinsinger R.F., Shirk M.C., Fall R. (2003). Rapid surface motility in *Bacillus subtilis* is dependent on extracellular surfactin and potassium ion. J. Bacteriol. Sep..

[36] Fall R., Kearns D.B., Nguyen T. (2006). A defined medium to investigate sliding motility in a *Bacillus subtilis* flagella-less mutant. BMC Microbial..

[37] Rashid M.H., Kornberg A. (2000). Inorganic polyphosphate is needed for swimming, swarming, and twitching motilities of *Pseudomonas aeruginosa*. Proc. Natl. Acad. Sci. USA.

[38] Ho T.H., Chuang C.Y., Zheng J.L., Chen H.H., Liang Y.S., Huang T.P., Lin Y.H. (2020). *Bacillus amyloliquefaciens* strain PMB05 intensifies plant immune responses to confer resistance against bacterial wilt of tomato. Phytopathology.

